# The impact of anti-bullying laws on children’s social-behavioral skills

**DOI:** 10.3389/fpsyg.2025.1550736

**Published:** 2025-04-28

**Authors:** Xiaohan Sun, Hang Zhao

**Affiliations:** ^1^The Business and Economics Department, Allegheny College, Meadville, PA, United States; ^2^The Computer and Information Science Department, Institutional Effectiveness, Allegheny College, Meadville, PA, United States

**Keywords:** school bullying, anti-bullying laws, social-behavioral skills, educational disparities, policy effectiveness

## Abstract

Bullying and violence, both on and off campuses, significantly impact children’s well-being. To address school bullying, every U.S. state gradually developed and implemented school anti-bullying laws (ABLs) and regulations between 2000 and 2015. This paper evaluates the effectiveness of ABLs using a difference-in-differences model and nationally representative samples of U.S. elementary school children. While state ABLs show limited overall effects on children’s social-behavioral skills, significant improvements are observed in self-control and interpersonal skills among low-income children, along with reduced externalizing behaviors among Hispanic children. States with strong or moderate ABLs show greater improvements in children’s interpersonal skills compared to states with weaker policies. These findings indicate social disparities in school bullying outcomes and highlight the importance of stronger policy enforcement.

## 1 Introduction

School bullying and violence remain critical social concerns worldwide, significantly impacting children’s mental health and personality development, and educational achievements (Robers et al., 2013; Puhl and King, 2013; Tang-Péronard and Heitmann, 2008; Davis et al., 2018; Datar and Sturm, 2006; Janssen et al., 2004; Puhl and Luedicke, 2012). Research consistently indicates the negative impact of bullying, including lower academic performance, higher dropout rates, and increased risk of suicide among victims (Puhl and King, 2013; Datar and Sturm, 2006; Puhl and Luedicke, 2012). Given these severe consequences, policymakers and researchers have emphasized the importance of effective bullying prevention strategies.

**Figure 1:**
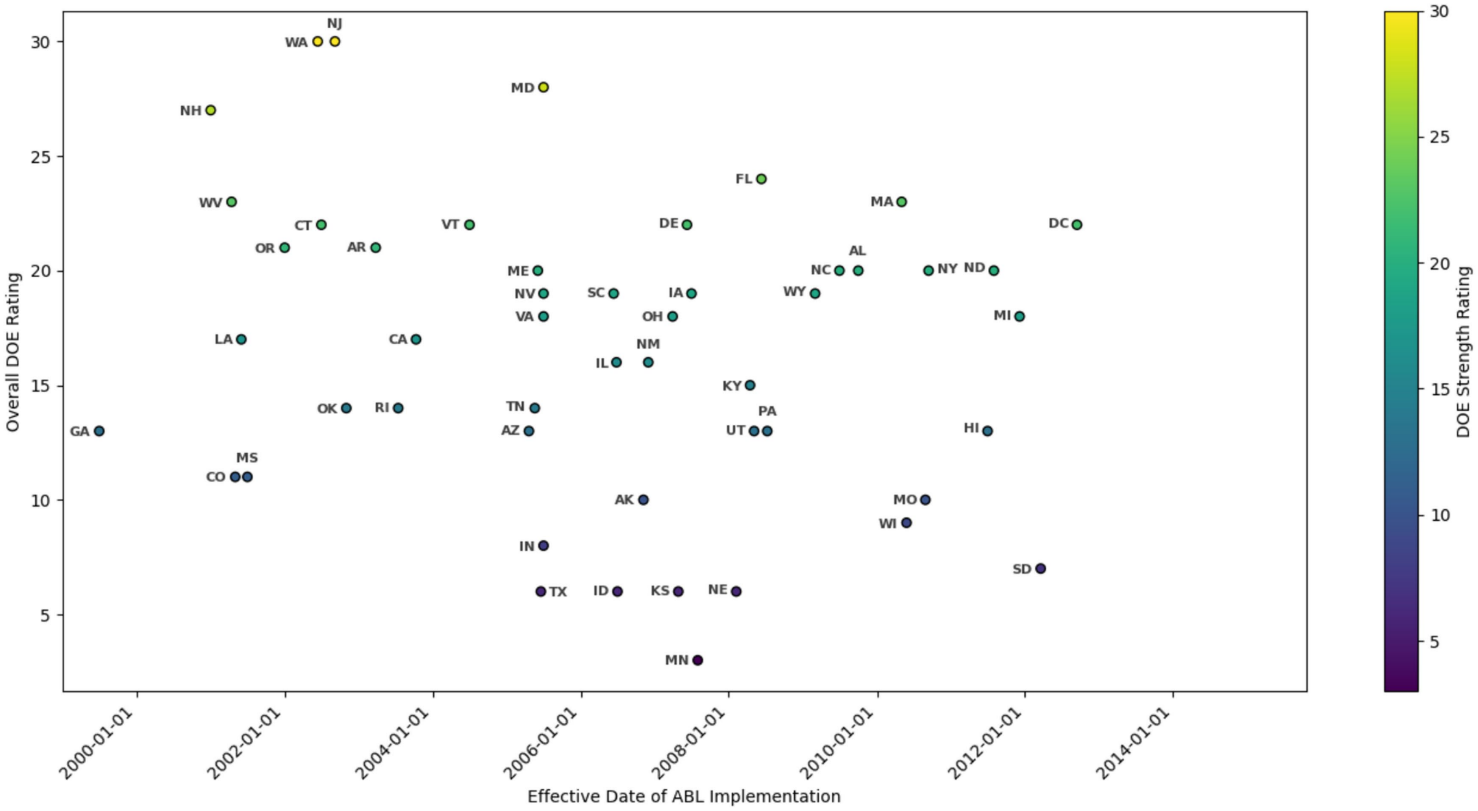
State anti-bullying policies effective dates and overall DOE rating. DOE: Department of Education. SOURCE: Effective dates: Nikolaou (2017). The General Assembly of each state. House Bill (H.B.), House File (H.F.), House Paper (H.P.), Senate Bill (S.B.), Assembly Bill (A.B.), Legislative Document (L.D.), Legislative Bill (L.B.). Overall DOE rating: Sabia and Bass (2017).

In response to growing concerns about school bullying, all U.S. states gradually developed and implemented school-based anti-bullying laws (ABLs) between 2000 and 2015 (Nikolaou, 2017). Georgia enacted the first law in 2000, and by 2015, Montana became the final state to adopt ABLs, ensuring nationwide coverage. However, the strength, strictness, and implementation timelines of these laws vary significantly across states. The U.S. Department of Education (DOE) evaluates state ABLs based on 16 legislative and policy components covering definitions, district policy development, district policy components, communication strategies, training, prevention measures, transparency, monitoring, and legal remedies (Sabia and Bass, 2017; Stuart-Cassel et al., 2011). As illustrated in Table 1 and Figure 1, these ratings classify state ABLs as weak, moderate, or strong, reflecting heterogeneity in anti-bullying policies across the nation.

**Table 1:** State anti-bullying policies effective dates and overall DOE rating.

State	Effective date	Overall DOE rating	State	Effective date	Overall DOE rating
Alabama	10/01/2009	20	Montana	04/21/2015	–
Alaska	11/06/2006	10	Nebraska	02/07/2008	6
Arizona	04/20/2005	13	Nevada	07/01/2005	19
Arkansas	03/26/2003	21	New Hampshire	01/01/2001	27
California	10/11/2003	17	New Jersey	09/06/2002	30
Colorado	05/02/2001	11	New Mexico	11/30/2006	16
Connecticut	07/01/2002	22	New York	09/13/2010	20
Delaware	06/09/2007	22	North Carolina	06/30/2009	20
District of Columbia	09/14/2012	22	North Dakota	08/01/2011	20
Florida	06/10/2008	24	Ohio	03/30/2007	18
Georgia	07/01/1999	13	Oklahoma	11/01/2002	14
Hawaii	07/01/2011	13	Oregon	01/01/2002	21
Idaho	07/01/2006	6	Pennsylvania	07/09/2008	13
Illinois	06/26/2006	16	Rhode Island	07/15/2003	14
Indiana	07/01/2005	8	South Carolina	06/12/2006	19
Iowa	07/01/2007	19	South Dakota	03/19/2012	7
Kansas	04/27/2007	6	Tennessee	05/19/2005	14
Kentucky	04/15/2008	15	Texas	06/18/2005	6
Louisiana	06/01/2001	17	Utah	05/05/2008	13
Maine	06/03/2005	20	Vermont	07/01/2004	22
Maryland	07/01/2005	28	Virginia	07/01/2005	18
Massachusetts	05/03/2010	23	Washington	06/13/2002	30
Michigan	12/06/2011	18	West Virginia	04/14/2001	23
Minnesota	08/01/2007	3	Wisconsin	05/27/2010	9
Mississippi	07/01/2001	11	Wyoming	03/02/2009	19
Missouri	08/28/2010	10			

DOE: Department of Education. Source: Effective dates: Nikolaou (2017). The General Assembly of each state. House Bill (H.B.), House File (H.F.), House Paper (H.P.), Senate Bill (S.B.), Assembly Bill (A.B.), Legislative Document (L.D.), Legislative Bill (L.B.). Overall DOE rating: Sabia and Bass (2017).

Given the widespread adoption of ABLs, a critical next step is to assess their effectiveness. Ideally, implementation of ABLs should reduce bullying by increasing the perceived cost and consequences for perpetrators, thus enhancing school safety and student well-being. However, existing literature provides mixed findings. While some studies show that ABLs reduce the frequency of bullying and victimization (Nikolaou, 2017; Seelman and Walker, 2018), others find minimal or no significant impact on school safety, mental health, or even increased bullying rates (Sabia and Bass, 2017; Seelman and Walker, 2018; Woods and Wolke, 2003). Due to controversial findings in prior literature, it is important to clearly understand the actual impact of ABLs.

Moreover, despite growing evidence indicating bullying occurs increasingly among younger children (Jansen et al., 2012), most prior studies have primarily focused on adolescents (Puhl and Luedicke, 2012; Nikolaou, 2017; Sabia and Bass, 2017; Seelman and Walker, 2018; Hatzenbuehler and Keyes, 2013). Consequently, there remains a substantial research gap regarding the effectiveness of ABLs on elementary school-aged children’s social-behavioral development. This study addresses this gap by evaluating the impact of state ABLs on social-behavioral skills among U.S. elementary school children using nationally representative data from the Early Childhood Longitudinal Study, Kindergarten Class of 1998–99 (ECLS-K).

Beyond academic achievements, the development of children’s social-behavioral skills has gained increasing attention from parents, educators, and policymakers due to their crucial role in lifelong well-being and success (Gottfried, 1985; Heckman and Rubinstein, 2001; Deke and Haimson, 2006; Heckman et al., 2006; Cunha and Heckman, 2008; Gottfried et al., 2011; Hill et al., 2011). ABLs, through targeted legal protections and clear behavioral expectations, could theoretically reduce bullying incidents, thus fostering improved social-behavioral outcomes among young children. However, existing evidence does not clearly indicate whether ABLs lead to measurable improvements in these skills and whether their impact varies by socioeconomic factors such as family income, ethnicity, or gender.

Based on the fact that different states have different ABLs effective date, we use a Difference-in-Difference (DID) model with child-specific random effects, state fixed effects, year fixed effects, and a range of child, family, school, and state control variables. We also include subgroup analyses to evaluate potential heterogeneous effects of ABLs, including by child’s poverty, ethnicity, and gender. We also include the DOE ratings in our analysis to distinguish between strong, moderate, and weak ABLs, enabling us to assess how policy strictness correlates with outcomes.

In summary, this study aims to contribute to existing literature and policy discussions by addressing the following research questions:

1. Do state anti-bullying laws (ABLs) improve children’s social-behavioral skills among elementary school children?
2. How do the effects of ABLs vary across different subgroups, particularly by income level, ethnicity, gender, and the strength of law enforcement?

## 2 Methods

### 2.1 Participants and procedure

The study is based on nationally representative samples of U.S. children in the Early Childhood Longitudinal Study, Kindergarten Class of 1998–99 (ECLS-K). The dataset offers ideal observational longitudinal data and focuses on children’s educational experiences from kindergarten through the 8th grade (National Center for Education Statistics, n.d.).

Parent, school, and child-level data were collected during multiple time points, including the fall and spring of kindergarten (1998–99), the fall and spring of 1st grade (1999–2000), the spring of 3rd grade (2002), and the spring of 5th grade (2004). The ECLS-K data cover 44 states and the District of Columbia for the period 1998–2007. The data cover children whose teachers completed social-behavioral skills questions for about 18,000 observations in the fall and spring of kindergarten, 15,000 observations in Grade 1, 12,000 observations in Grade 3, and 11,000 observations in Grade 5. According to the regulations for restricted-use data of the Institute of Education Sciences Data Security Office, all sample sizes and degrees of freedom in this paper are rounded to the nearest 50.

### 2.2 Measures

#### 2.2.1 Dependent variables

Behavioral and social skills are measured by scales rated by teachers that were collected in fall 1998, spring 1999, spring 2000, spring 2002, and spring 2004.

For behavioral skills, two scales ranging from 1 to 4 related to students’ problem behaviors, including externalizing the problem (arguing, fighting, getting angry, acting impulsively, and disturbing ongoing activities) and internalizing the problem (anxiety, loneliness, low self-esteem, and sadness). Lower scores are favorable for the externalizing problem scale and internalizing problem scale, since a lower score indicates that the teacher reported a low frequency of problem behaviors for the given child (Datar and Gottfried, 2015; Pollack et al., 2005).

Social skills are measured by three scales ranging from 1 to 4, including approaches to learning (attentiveness, task persistence, eagerness to learn, learning independence, flexibility, and organization); self-control (controlling temper, accepting peer ideas, respecting others’ property, and handling peer pressure); and interpersonal skills (getting along with others; forming and maintaining friendships; helping other children; expressing feelings, ideas, and opinions in positive ways; and showing sensitivity to the feelings of others). Higher scores are favorable for the three scales, since a higher score indicates that the student was observed to exhibit more of the skills described above on the teacher’s report (Datar and Gottfried, 2015; Pollack et al., 2005).

#### 2.2.2 Explanatory variables

The primary independent variable is a dichotomous measure of whether a state has implemented an ABL in that year based on the ABL’s effective date in Table 1 and Figure 1. To test the strength of law enforcement and the laws’ effectiveness, we generate three indicators based on the DOE rating of ABLs. According to the thresholds in Sabia and Bass (2017), a strong ABL equals 1 if the state’s overall DOE rating of ABLs is in the upper 25th percentile (between 21st and 32nd) and 0 otherwise; a moderate ABL equals 1 if the state’s overall DOE rating of ABLs is in the 25th to 75th percentiles (between 13 and 20) and 0 otherwise; a weak ABL equals 1 if the state’s overall DOE rating of ABLs is in the lower 25th percentile (between 0 and 12) and 0 otherwise (Sabia and Bass, 2017). The classification of weak, moderate, and strong ABLs follows Sabia and Bass (2017), based on the U.S. Department of Education (DOE) ratings. These thresholds align with policy variations across states and reflect meaningful distinctions in ABL strength. Given their established robustness, we adopt this classification in our analysis. Other explanatory variables are child, family, and school characteristics, including child’s gender, race, poverty status, family income, number of siblings, mother’s education, school size, percentage of minorities in the school, private school, percentage of students eligible for a free lunch, and geographic region. We also control for time-varying state characteristics, such as the state unemployment rate (United States Department of Labor) and state per capita income (Federal Reserve Bank of St. Louis). A measure for the duration of exposure to the ABL was also added as a covariate.

Descriptive statistics for all variables are reported in Table 2. It shows that the mean scale of externalizing problem behaviors is 1.659, the mean scale of internalizing problem behaviors is 1.588, the mean scale of approaches to learning is 3.041, the mean scale of self-control is 3.156, and the mean scale of interpersonal skills is 3.062.

**Table 2:** Descriptive statistics of outcomes and control variables, by data source.

	Mean (SE)	Source
Outcomes		
Externalizing Problem Behaviors scale	1.659 (0.006) [*N* = 55,300]	ECLS-K
Internalizing Problem Behaviors scale	1.588 (0.002) [*N* = 54,850]	ECLS-K
Approaches to Learning scale	3.041 (0.003) [*N* = 55,650]	ECLS-K
Self-Control scale	3.156 (0.002) [*N* = 54,800]	ECLS-K
Interpersonal Skills scale	3.062 (0.002) [*N* = 54,400]	ECLS-K
Anti-bullying laws		
ABL	0.172 (0.001)	ECLS-K
Strong ABL	0.020 (0.0004)	ECLS-K and Department of Education
Moderate ABL	0.119 (0.001)	ECLS-K and Department of Education
Weak ABL	0.032 (0.001)	ECLS-K and Department of Education
Demographic controls		
Male	0.512 (0.001)	ECLS-K
White	0.664 (0.001)	ECLS-K
Black	0.164 (0.001)	ECLS-K
Hispanic	0.178 (0.001)	ECLS-K
Asian	0.069 (0.001)	ECLS-K
Below poverty threshold	0.210 (0.001)	ECLS-K
Family income (<US$5,000)	0.032 (0.001)	ECLS-K
Number of siblings in household	1.521 (0.003)	ECLS-K
Mother’s education: less than high school	0.123 (0.001)	ECLS-K
School size (0–149)	0.062 (0.001)	ECLS-K
Less than 10% minority in school	0.280 (0.001)	ECLS-K
Private school	0.175 (0.001)	ECLS-K
Percentage of students eligible for free lunch	32.740 (0.075)	ECLS-K
Northeast	0.184 (0.001)	ECLS-K
Midwest	0.248 (0.001)	ECLS-K
South	0.334 (0.001)	ECLS-K
West	0.235 (0.001)	ECLS-K
Duration of ABLs exposure	0.471 (0.004)	ECLS-K
State-specific economic controls		
Unemployment rate	4.755 (0.009)	USDL
Per capita personal income	31658 (49.125)	St. Louis Fed

Source: U.S. Department of Education, National Center for Education Statistics, Early Childhood Longitudinal Study, Kindergarten Class of 1998–99 (ECLS-K), fall 1998, spring 1999, fall 1999, spring 2000, spring 2002, and spring 2004. ELCS-K: The Early Childhood Longitudinal Study, Kindergarten Class of 1998–99 (ECLS-K). USDL, United States Department of Labor; ST. Louis Fed, Federal Reserve Bank of St. Louis; ABL, anti-bullying laws; N reports child-year observations. According to the regulations for restricted-use data produced by the Institute of Education Sciences Data Security Office, all the sample sizes and degrees of freedom in this paper are rounded to the nearest 50.

### 2.3 Data analysis

There is a fundamental identification problem in a simple comparison of social-behavioral skills outcomes before and after the introduction of ABLs, since these outcomes are not time-invariant and can change over time irrespective of any policy. Based on the NCES report, the incidence of school violence and bullying has increased in recent years (Robers et al., 2013). In addition, children’s social skills and behavioral skills have changed over time due to factors such as their weight and family environment (Puhl and King, 2013; Tang-Péronard and Heitmann, 2008). As a result, the increase or decrease in outcome variables after one state’s introduction of ABLs might not represent the real effect of ABLs. Based on variation in the effective date of each state’s ABLs, we use a linear DID method to identify causality.

Under the assumption that the decision of state ABLs effective date is random, $\beta_{1}$ in the difference-in-difference model captures causal estimates of the effects of ABLs. The model is specified as:


(1) $$Y_{jst}=\beta_{0}+\beta_{1}\mathrm{AB}L_{st}+\beta_{2}X_{jst}+\beta_{3}Z_{st}+\mu_{s}+\gamma_{t}+\pi_{j}+\varepsilon_{jst}$$

where $Y_{jst}$ is the outcome variable for student j in state s in year t, $\mathrm{AB}L_{st}$ is a dichotomous measure of whether state s has an effective ABL in year t, $X_{jst}$ is other explanatory variables related to child, family, and school characteristics for student j in states in year t, and $Z_{st}$ controls for state time-varying characteristics during the implementation of an ABL to isolate economic conditions that might affect the outcomes, such as state unemployment and per capita income. $\mu_{s}$ represents a state fixed effect controlling for state time-invariant characteristics, $\gamma_{t}$ represents year fixed effects $,\pi_{j}$ represents a child-specific random effect to control for time-invariant heterogeneity at child level, and $\varepsilon_{jst}$ is the error term.

We use a child-specific random-effects model instead of a fixed-effects model, as random effects capture variation across individuals while accounting for unobserved differences without absorbing within-child variation (e.g., ethnicity, gender, and race, which remain constant over time). Since our focus is on policy effects across different groups, the random-effects approach is more appropriate. To address potential endogeneity and mitigate omitted variable concerns, we conducted a Hausman test, which confirmed that the random-effects model does not introduce significant bias compared to the fixed-effects model, validating our choice.

The study includes several subgroup analyses to evaluate the heterogeneous effects of ABLs, including by child’s poverty, ethnicity, and gender, and a subsample of only strong or moderate ABLs that excludes weak ABLs to test for policy heterogeneity due to the strictness of state ABLs.

### 2.4 Validity of the difference-in-difference model

The key assumption of the DID model is the parallel trends assumption, which implies that in the absence of the policy, the trends of change in the control group were the same as the trends of change in the treatment group (Li et al., 2012). For example, if outcomes in the treatment group were improving faster than in the control group before the introduction of ABLs, the DID model would overestimate the effect of ABLs. This study tests the parallel trends assumption using an event study framework to demonstrate the robustness of the DID model, which is consistent with prior economic research on ABLs’ effects (Sabia and Bass, 2017). Specifically, the model in Equation 1 was re-estimated by including a set of indicators to capture the years before and after the introduction of ABLs. The leads and lags of ABLs indictors include 1 year prior to introduction of the policy, 2 years prior, 3 years prior, 4 or more years prior, 1 year after introduction of the policy, 2 years after, and 3 or more years after. The statistical insignificance of the lagged treatment indicators shown in the panels for the parallel trends test in Tables 3–7 indicate that the trends for the control and treatment group are the same before the policy intervention. Thus, the DID model can yield a causal interpretation of the effect of ABLs. While some event study results show minor pre-trends in certain outcomes, these fluctuations are small and inconsistent across models. Given that the core post-policy effects remain robust and statistically significant, we conclude that the parallel trends assumption holds sufficiently for causal inference.

**Table 3:** The effects of anti-bullying laws on children’s social-behavioral skills.

	Externalizing problem behaviors scale (1)	Internalizing problem behaviors scale (2)	Approaches to learning scale (3)	Self-control scale (4)	Interpersonal skills scale (5)
ABL	0.011	−0.006	−0.013	0.006	0.023
	(0.016)	(0.017)	(0.018)	(0.018)	(0.019)
Parallel Trend Test: Leads and lags of ABL					
4 or more years before	−0.022	−0.02	−0.032*	−0.027*	−0.022
	(0.012)	(0.012)	(0.014)	(0.013)	(0.014)
3 years before	0.001	−0.024	−0.002	−0.008	−0.008
	(0.010)	(0.011)	(0.012)	(0.012)	(0.013)
2 years before	−0.024	−0.038**	−0.0002	−0.003	−0.001
	(0.013)	(0.014)	(0.015)	(0.015)	(0.016)
1 year before	–	–	–	–	–
Year of accepting	0.005	0.012	0.002	0.028	0.016
	(0.018)	(0.019)	(0.020)	(0.019)	(0.021)
1 year after	0.018	−0.006	0.056	0.014	0.019
	(0.027)	(0.025)	(0.029)	(0.029)	(0.030)
2 years later	0.077	0.056	0.087	−0.006	−0.03
	(0.053)	(0.051)	(0.061)	(0.057)	(0.059)
3 or more years later	0.06	0.047	0.246**	0.040	0.006
	(0.078)	(0.074)	(0.087)	(0.084)	(0.086)
Observations	55,300	54,850	55,650	54,800	54,400

SOURCE: U.S. Department of Education, National Center for Education Statistics, Early Childhood Longitudinal Study, Kindergarten Class of 1998–99 (ECLS-K), fall 1998, spring 1999, fall 1999, spring 2000, spring 2002, and spring 2004. Figures in parentheses are the respective robust standard errors. ***p < 0.001, **p < 0.01, *p < 0.05. ABL: Anti-bullying Laws. 1 year before is the reference category. N reports child-year observations. All regressions include child-specific random effects, year fixed effects, state fixed effects, and other explanatory variables related to child, family, school characteristics including child’s gender, race, poverty status, family income, number of siblings, mother’s education, school size, percentage of minority in school, private school, percentage of students eligible for free lunch, and geographic region, and time-varying state characteristics including state unemployment rate and state per capita income, and a measure of duration of ABLs exposure. According to the regulations for restricted-use data produced by the Institute of Education Sciences Data Security Office, all the sample sizes and degrees of freedom in this paper are rounded to the nearest 50.

**Table 4:** Heterogeneous Effects of Anti-bullying Laws by Poverty Level.

	Externalizing problem behaviors scale (1)	Internalizing problem behaviors scale (2)	Approaches to learning scale (3)	Self-control scale (4)	Interpersonal skills scale (5)
Panel A: Below poverty threshold					
ABL	−0.062	−0.041	0.066	0.117**	0.086*
	(0.036)	(0.041)	(0.039)	(0.038)	(0.039)
Parallel trend test: leads and lags of ABL					
4 or more years before	−0.040	−0.047	−0.044	−0.013	−0.018
	(0.031)	(0.032)	(0.034)	(0.034)	(0.035)
3 years before	−0.023	−0.050	0.020	0.015	0.005
	(0.025)	(0.028)	(0.028)	(0.029)	(0.030)
2 years before	−0.067*	−0.043	0.016	0.027	0.032
	(0.032)	(0.034)	(0.036)	(0.035)	(0.036)
1 year before	–	–	–	–	–
Year of accepting	−0.045	0.047	0.041	0.121**	0.071
	(0.051)	(0.052)	(0.049)	(0.046)	(0.051)
1 year after	−0.035	−0.040	0.146*	0.107	0.071
	(0.053)	(0.066)	(0.060)	(0.056)	(0.059)
2 years later	0.003	0.121	0.244	0.152	0.074
	(0.108)	(0.140)	(0.143)	(0.125)	(0.139)
3 or more years later	0.090	0.047	0.324	0.045	0.008
	(0.154)	(0.191)	(0.168)	(0.159)	(0.170)
Observations	10,250	10,100	10,300	10,100	10,000
Panel B: At or above poverty threshold					
ABL	0.028	−0.001	−0.035	−0.024	0.006
	(0.018)	(0.018)	(0.020)	(0.020)	(0.021)
Parallel trend test: leads and lags of ABL					
4 or more years before	−0.012	−0.010	−0.034*	−0.036*	−0.027
	(0.013)	(0.013)	(0.015)	(0.014)	(0.015)
3 years before	0.012	−0.016	−0.010	−0.018	−0.013
	(0.011)	(0.012)	(0.013)	(0.013)	(0.014)
2 years before	−0.010	−0.034*	−0.008	−0.018	−0.016
	(0.014)	(0.015)	(0.017)	(0.016)	(0.018)
1 year before	–	–	–	–	–
Year of accepting	0.018	0.008	−0.008	0.005	0.003
	(0.019)	(0.020)	(0.022)	(0.021)	(0.023)
1 year after	0.031	−0.001	0.035	−0.009	0.012
	(0.030)	(0.027)	(0.033)	(0.033)	(0.034)
2 years later	0.090	0.047	0.056	−0.037	−0.050
	(0.061)	(0.054)	(0.068)	(0.065)	(0.066)
3 or more years later	0.045	0.040	0.245*	0.054	0.038
	(0.089)	(0.078)	(0.101)	(0.097)	(0.099)
Observations	45,050	44,800	45,300	44,650	44,400

Source: U.S. Department of Education, National Center for Education Statistics, Early Childhood Longitudinal Study, Kindergarten Class of 1998–99 (ECLS-K), fall 1998, spring 1999, fall 1999, spring 2000, spring 2002, and spring 2004. Figures in parentheses are the respective robust standard errors. ***p < 0.001, **p < 0.01, *p < 0.05. ABL: Anti-bullying Laws. 1 year before is the reference category. N reports child-year observations. All regressions include child-specific random effects, year fixed effects, state fixed effects, and other explanatory variables related to child, family, school characteristics including child’s gender, race, poverty status, family income, number of siblings, mother’s education, school size, percentage of minority in school, private school, percentage of students eligible for free lunch, and geographic region, and time-varying state characteristics including state unemployment rate and state per capita income, and a measure of duration of ABLs exposure. According to the regulations for restricted-use data produced by the Institute of Education Sciences Data Security Office, all the sample sizes and degrees of freedom in this paper are rounded to the nearest 50.

**Table 5:** Heterogeneous effects of anti-bullying laws by ethnicity.

	Externalizing problem behaviors scale (1)	Internalizing problem behaviors scale (2)	Approaches to learning scale (3)	Self-control scale (4)	Interpersonal skills scale (5)
Panel A: Hispanic					
ABL	−0.085*	−0.053	−0.009	0.071	0.012
	(0.040)	(0.047)	(0.047)	(0.047)	(0.050)
Parallel trend test: leads and lags of ABL					
4 or more years before	−0.050	−0.023	0.005	−0.008	0.021
	(0.029)	(0.029)	(0.033)	(0.032)	(0.033)
3 years before	−0.003	0.01	0.029	0.004	0.013
	(0.024)	(0.026)	(0.029)	(0.028)	(0.029)
2 years before	−0.062	−0.032	0.044	0.050	0.022
	(0.036)	(0.042)	(0.048)	(0.044)	(0.046)
1 year before	–	–	–	–	–
Year of accepting	−0.022	0.009	0.006	0.109*	0.122
	(0.054)	(0.066)	(0.057)	(0.053)	(0.065)
1 year after	−0.087	0.069	0.045	0.127	−0.074
	(0.079)	(0.069)	(0.073)	(0.090)	(0.069)
2 years later	−0.053	0.226	0.116	0.144	−0.256
	(0.164)	(0.145)	(0.155)	(0.186)	(0.153)
3 or more years later	−0.066	0.356	0.139	0.277	−0.256
	(0.247)	(0.242)	(0.242)	(0.291)	(0.228)
Observations	9,400	9,300	9,500	9,250	9,150
Panel B: Not Hispanic					
ABL	0.032	0.006	−0.020	−0.013	0.017
	(0.018)	(0.019)	(0.020)	(0.020)	(0.021)
Parallel trend test: leads and lags of ABL					
4 or more years before	−0.014	−0.026	−0.035*	−0.028	−0.022
	(0.013)	(0.014)	(0.015)	(0.015)	(0.016)
3 years before	0.006	−0.036**	−0.007	−0.006	−0.009
	(0.012)	(0.013)	(0.014)	(0.013)	(0.014)
2 years before	−0.014	−0.043**	−0.005	−0.008	−0.003
	(0.014)	(0.015)	(0.017)	(0.016)	(0.017)
1 year before	–	–	–	–	–
Year of accepting	0.015	0.010	0.0003	0.019	0.006
	(0.019)	(0.020)	(0.021)	(0.021)	(0.023)
1 year after	0.040	−0.006	0.049	−0.004	0.016
	(0.028)	(0.027)	(0.031)	(0.031)	(0.032)
2 years later	0.094	0.042	0.073	−0.010	−0.021
	(0.055)	(0.053)	(0.063)	(0.060)	(0.062)
3 or more years later	0.076	0.029	0.246**	0.027	0.013
	(0.081)	(0.076)	(0.090)	(0.087)	(0.090)
Observations	45,900	45,550	46,150	45,550	45,250

Source: U.S. Department of Education, National Center for Education Statistics, Early Childhood Longitudinal Study, Kindergarten Class of 1998–99 (ECLS-K), fall 1998, spring 1999, fall 1999, spring 2000, spring 2002, and spring 2004. Figures in parentheses are the respective robust standard errors. ***p < 0.001, **p < 0.01, *p < 0.05. ABL: Anti-bullying Laws. 1 year before is the reference category. N reports child-year observations. All regressions include child-specific random effects, year fixed effects, state fixed effects, and other explanatory variables related to child, family, school characteristics including child’s gender, race, poverty status, family income, number of siblings, mother’s education, school size, percentage of minority in school, private school, percentage of students eligible for free lunch, and geographic region, and time-varying state characteristics including state unemployment rate and state per capita income, and a measure of duration of ABLs exposure. According to the regulations for restricted-use data produced by the Institute of Education Sciences Data Security Office, all the sample sizes and degrees of freedom in this paper are rounded to the nearest 50.

**Table 6:** Heterogeneous effects of anti-bullying laws by gender.

	Externalizing problem behaviors scale (1)	Internalizing problem behaviors scale (2)	Approaches to learning scale (3)	Self-control scale (4)	Interpersonal skills scale (5)
Panel A: Female					
ABL	−0.016	−0.016	−0.006	0.013	0.030
	(0.020)	(0.023)	(0.024)	(0.024)	(0.026)
Parallel trend test: leads and lags of ABL					
4 or more years before	−0.004	−0.027	−0.021	−0.033	−0.001
	(0.015)	(0.017)	(0.019)	(0.018)	(0.019)
3 years before	0.021	−0.020	−0.007	−0.015	0.009
	(0.013)	(0.016)	(0.016)	(0.016)	(0.018)
2 years before	0.003	−0.035	−0.007	−0.019	0.008
	(0.017)	(0.020)	(0.021)	(0.020)	(0.022)
1 year before	–	–	–	–	–
Year of accepting	0.008	0.018	0.002	0.035	0.056
	(0.023)	(0.026)	(0.027)	(0.026)	(0.029)
1 year after	0.024	−0.019	0.043	−0.009	0.027
	(0.035)	(0.035)	(0.039)	(0.040)	(0.041)
2 years later	0.117	0.036	0.079	−0.020	−0.009
	(0.070)	(0.070)	(0.080)	(0.081)	(0.082)
3 or more years later	0.125	0.021	0.192	−0.046	−0.006
	(0.103)	(0.099)	(0.116)	(0.119)	(0.121)
Observations	27,500	27,350	27,700	27,250	27,200
Panel B: Male					
ABL	0.038	0.003	−0.021	−0.003	0.016
	(0.025)	(0.025)	(0.026)	(0.026)	(0.027)
Parallel trend test: leads and lags of ABL					
4 or more years before	−0.041*	−0.013	−0.043*	−0.020	−0.044*
	(0.018)	(0.018)	(0.020)	(0.019)	(0.020)
3 years before	−0.018	−0.028	0.002	−0.001	−0.026
	(0.016)	(0.016)	(0.018)	(0.017)	(0.018)
2 years before	−0.049*	−0.041*	0.006	0.012	−0.013
	(0.020)	(0.020)	(0.022)	(0.022)	(0.023)
1 year before	–	–	–	–	–
Year of accepting	−0.0002	0.005	0.004	0.023	−0.021
	(0.026)	(0.027)	(0.029)	(0.029)	(0.031)
1 year after	0.011	0.008	0.070	0.038	0.009
	(0.041)	(0.037)	(0.044)	(0.040)	(0.042)
2 years later	0.029	0.079	0.111	0.017	−0.043
	(0.080)	(0.074)	(0.091)	(0.080)	(0.084)
3 or more years later	−0.014	0.078	0.310*	0.131	0.021
	(0.119)	(0.109)	(0.131)	(0.115)	(0.123)
Observations	27,750	27,550	27,950	27,550	27,250

Source: U.S. Department of Education, National Center for Education Statistics, Early Childhood Longitudinal Study, Kindergarten Class of 1998–99 (ECLS-K), fall 1998, spring 1999, fall 1999, spring 2000, spring 2002, and spring 2004. Figures in parentheses are the respective robust standard errors. ***p < 0.001, **p < 0.01, *p < 0.05. ABL: Anti-bullying Laws. 1 year before is the reference category. N reports child-year observations. All regressions include child-specific random effects, year fixed effects, state fixed effects, and other explanatory variables related to child, family, school characteristics including child’s gender, race, poverty status, family income, number of siblings, mother’s education, school size, percentage of minority in school, private school, percentage of students eligible for free lunch, and geographic region, and time-varying state characteristics including state unemployment rate and state per capita income, and a measure of duration of ABLs exposure. According to the regulations for restricted-use data produced by the Institute of Education Sciences Data Security Office, all the sample sizes and degrees of freedom in this paper are rounded to the nearest 50.

**Table 7:** The effects of anti-bullying laws on children’s social-behavioral skills, strong or moderate ABLs.

	Externalizing problem behaviors scale (1)	Internalizing problem behaviors scale (2)	Approaches to Learning scale (3)	Self-control scale (4)	Interpersonal skills scale (5)
ABL	−0.009	−0.025	0.010	0.016	0.046*
	(0.017)	(0.019)	(0.020)	(0.020)	(0.021)
Parallel trend test: leads and lags of ABL					
4 or more years before	−0.023	−0.020	−0.031*	−0.026*	−0.020
	(0.012)	(0.012)	(0.014)	(0.013)	(0.014)
3 years before	0.002	−0.023*	−0.001	−0.007	−0.007
	(0.010)	(0.011)	(0.012)	(0.012)	(0.013)
2 years before	−0.022	−0.038**	0.003	−0.002	−0.0004
	(0.013)	(0.014)	(0.015)	(0.015)	(0.016)
1 year before	–	–	–	–	–
Year of accepting	0.005	0.013	−0.003	0.026	0.012
	(0.018)	(0.019)	(0.020)	(0.019)	(0.021)
1 year after	0.010	−0.011	0.030	0.006	0.002
	(0.028)	(0.026)	(0.031)	(0.030)	(0.031)
2 years later	0.090	0.074	0.036	−0.027	−0.076
	(0.054)	(0.052)	(0.062)	(0.059)	(0.061)
3 or more years later	0.095	0.092	0.099	−0.020	−0.129
	(0.084)	(0.081)	(0.095)	(0.090)	(0.095)
Observations	54,600	54,250	54,950	54,100	53,750

Source: U.S. Department of Education, National Center for Education Statistics, Early Childhood Longitudinal Study, Kindergarten Class of 1998–99 (ECLS-K), fall 1998, spring 1999, fall 1999, spring 2000, spring 2002, and spring 2004. Figures in parentheses are the respective robust standard errors. ***p < 0.001, **p < 0.01, *p < 0.05. ABL: Anti-bullying Laws. 1 year before is the reference category. N reports child-year observations. All regressions include child-specific random effects, year fixed effects, state fixed effects, and other explanatory variables related to child, family, school characteristics including child’s gender, race, poverty status, family income, number of siblings, mother’s education, school size, percentage of minority in school, private school, percentage of students eligible for free lunch, and geographic region, and time-varying state characteristics including state unemployment rate and state per capita income, and a measure of duration of ABLs exposure. According to the regulations for restricted-use data produced by the Institute of Education Sciences Data Security Office, all the sample sizes and degrees of freedom in this paper are rounded to the nearest 50.

## 3 Results

### 3.1 Effect of ABLs on children’s social-behavioral skills

Table 3 reports the effects of ABLs on the outcome variables. Estimates for externalizing problem behaviors scale outcomes are shown in Column (1), estimates for internalizing problem behaviors scale outcomes in Column (2), estimates for approaches to learning scale outcomes in Column (3), estimates for self-control scale outcomes in Column (4), and estimates for interpersonal skills scale outcomes in Column (5). The results in Table 3 show little evidence that ABLs have an effect on children’s social-behavioral skills for the whole sample.

### 3.2 Heterogeneous effects of ABLs

Tables 4–7 reports the heterogeneous effects of ABLs by child’s poverty, ethnicity, and gender, and a subsample of only strong or moderate ABLs that excludes weak ABLs.

Table 4 shows results separated by children living in households with income below the federal poverty threshold and children living in households with income at or above the threshold. The results in Panel A of Table 4, for children living in households with income below the threshold, show that the adoption of state ABLs is associated with a 0.117 scale points increase on the self-control scale and a 0.086 scale points increase on the interpersonal skills scale, which indicates a positive relationship between ABLs and these positive skills for children from less-advantaged families. The non-significant estimates in Panel B of Table 4 for children living in households with income at or above the threshold show that there is no effect of ABLs on children in high-income families, which indicates a larger and more significant effect on children in less-advantaged families.

Estimates of ABLs’ effect on outcome variables by ethnicity are shown in Table 5. Results are separated by those for Hispanic children and Non-Hispanic children. The results in Panel A of Table 5, for Hispanic children, show that the adoption of state ABLs is associated with a 0.085 scale points reduction on the externalizing problems behaviors scale. The reduction in externalizing problems behaviors is favorable, since it means that children exhibit fewer externalizing behavior problems. The non-significant estimates in Panel B of Table 5 for Non-Hispanic children show that there is no effect of ABLs on Non-Hispanic children.

Estimates of ABLs’ effect on outcome variables by gender are reported in Table 6, and the results show that there is no significant effect of ABLs for either female or male subgroups.

Table 7 reports the results for a subsample limited to strong or moderate ABLs only to test policy heterogeneity due to the strictness of state ABLs. Specifically, in the analysis of Table 7, we excluded states with weak ABLs whose overall DOE ABL rating is in the lower 25th percentile. The results in Table 7 shows that in the sample of states with strong or moderate ABLs, the adoption of strong or moderate ABLs is related to a 0.046 scale points increase on the interpersonal skills scale, which represents a positive relationship between ABLs and children’s interpersonal skills. Compared with the null finding for the full sample, the positive and significant results for strong policies indicate that the strength and strictness of state ABLs is associated with the effects of ABLs.

## 4 Discussion

The paper finds little evidence of the effect of state ABLs on children’s social-behavioral skills for the full sample. However, we find a significant positive effect of ABLs on the self-control scale and the interpersonal skills scale for children from less-advantaged families, and a significant reduction on the externalizing problem behaviors scale for Hispanic children. After restricting the strength and strictness of state ABLs, strong or moderate ABLs are associated with a significant improvement in children’s interpersonal skills.

Lacking statistical significance for the full sample might indicate that there is no effect of ABLs on children’s social-behavioral problems, which is consistent with much of the previous literature (Sabia and Bass, 2017; Seelman and Walker, 2018). However, the null finding could be related to potential unmeasured confounders, such as variations in enforcement at both state level and school level. While ABLs establish a legal framework, their actual implementation depends on local enforcement policies, school resources, and community engagement. Some states and school districts may actively implement and monitor compliance with ABLs, while others may lack the necessary resources or commitment, potentially weakening the overall impact observed in the study.

For data limitations, the outcomes in this paper are children’s social-behavioral skills and not direct school-bullying victimization. The school-bullying-victimization variable is only collected at two datapoints in the ECLS-K data, and is weak for panel data analysis and unsuitable for the DID model design. Additionally, the relatively wide confidence intervals (larger standard errors) in some subgroup analyses may be due to greater variability resulting from smaller sample sizes. While certain subgroups may have limited sample sizes, the overall sample remains substantial, supporting the robustness of the main findings.

Regarding the methodological limitation, there might be a mechanism whereby the validity conditions of the DID model are not met. For causal interpretation of the DID model, the assumption should be satisfied that states’ choice of an ABL’s effective date is random and not related to unobserved characteristics. However, the assumption could be violated, since states’ ABL implementation date might not be random and instead related to some state characteristics, such as the suicide rate or crime rate. In addition, the DID model might not reveal the true effect of ABLs. During the ABL implementation period, concurrent state policies or economic conditions could play a role and might also have an effect on the outcomes. Even though we control for some economic conditions, such as the state’s unemployment rate and per capita income, it is hard to control for all relevant policies in effect between 1998 and 2007.

## 5 Conclusion

This study is the first to examine the effects of state ABLs on children’s social-behavioral skills using nationally representative samples of younger U.S. children. The DID model indicates that there is no significant effect of ABLs on younger children’s social-behavioral skills for the full sample. However, by evaluating the heterogeneous effects by child’s characteristics and the strictness of ABL enforcement, we find that for children living in households with income below the federal poverty threshold, the adoption of state ABLs is associated with a 0.117 scale points increase on the self-control scale and a 0.086 scale points increase on the interpersonal skills scale, which indicates a positive relationship between ABLs and these positive skills for children living in households with income below the federal poverty threshold. Also, the adoption of state ABLs is associated with a 0.085 scale points reduction on the externalizing problem behaviors scale for Hispanic children. After limiting the analysis to states with strong or moderate ABLs, we find a 0.046 scale points increase on the interpersonal skills scale, which represents a positive relationship between strong or moderate ABLs and children’s interpersonal skills.

Our findings indicate that ABLs are more effective for children from less-advantaged families and Hispanic children. These results may suggest that children from low-income families and Hispanic children have a higher probability of being involved in school bullying and victimization (Jansen et al., 2012; Christie-Mizell, 2004). Schools serving more minority students and those in economically disadvantaged areas may have higher exposure to school bullying incidents. As a result, these schools have stronger incentives to implement stricter policies, which could explain the effective implementation of ABLs.

By distinguishing the strictness of state ABL enforcement, the study emphasizes the necessity of implementing strong ABLs, since the implementation of weak policy cannot be very effective. States that do not mandate strong ABLs should learn from other states in a timely manner, and improve and strengthen their own mandates. The purpose pf implementing ABLs is to reduce bullying and further improve children’s well-being, which is what all states hope to accomplish. Therefore, it is essential that states strengthen their communication, learn from each other, and implement strong ABLs.

The null findings in the whole sample could be also due to weak legal awareness on the part of young children and inadequate implementation of the law. The study, therefore, can provide a useful reference for further improvement of anti-bullying laws.

Nowadays, the high incidence of school bullying and its negative impact on the healthy development of children are indisputable, and this must be widely acknowledged by the society at large. It is important that laws and regulations for school bullying be improved.

We have the following recommendations to enhance the effectiveness of anti-bullying laws in schools. The first is to strengthen legal education and improve anti-bullying policy awareness among children, especially younger children. This is fundamental for preventing bullying. Communities, families, and schools should make use of their respective advantages, popularize relevant legal knowledge, and carry out bullying prevention education in a way that children will be receptive to and understand. For example, parents can use storytelling methods to inform school-age children of typical bullying cases that have occurred around them, instilling the importance of law-abiding behavior from a young age. Schools should incorporate special courses and appoint counselors to facilitate ongoing discussions about bullying prevention in every classroom. At the same time, anti-bullying education and guidance should be given to children, including seeking help when bullied, building friendships, engaging in physical fitness activities, and developing personal resilience. Cultivating children’s legal awareness should be the long-term plan for the whole society.

Even though our findings suggest that the strictness of ABLs may influence their effectiveness, policymakers should carefully consider the appropriate level of enforcement to avoid unintended consequences. Overly strict policies could create an overly intense school environment, potentially harming students’ academic outcomes or mental health (O'Malley et al., 2015). In particular, we want to avoid a situation where stricter enforcement of ABLs in economically disadvantaged areas, compared to more economically advantaged areas, could inadvertently widen social disparities.

## Data Availability

The data analyzed in this study is subject to the following licenses/restrictions: the dataset used in this study is the restricted-use version of the Early Childhood Longitudinal Study, Kindergarten Class of 1998-99 (ECLS-K), and is available through a licensing agreement with the Institute of Education Sciences (IES). All analyses comply with the IES Data Security Office requirements. As long as researchers apply for and receive approval to access the restricted version of the ECLS-K dataset, they should be able to replicate all the analyses in this paper. Requests to access these datasets should be directed to IES Data Security, IESData.Security@ed.gov.
